# Prevalence, incidence, and thromboembolic events in polycythemia vera: a study based on longitudinal German health claims data

**DOI:** 10.1007/s00277-025-06192-6

**Published:** 2025-02-10

**Authors:** Karina C. Manz, Anja Mocek, Bashar Morouj, Katharina Merker, Marc Feuerbach, Ariane Höer, Valeria Weber, Raeleesha Norris, Susanne Grosser, Frank Andersohn, Haifa Kathrin Al-Ali

**Affiliations:** 1 IGES Institut GmbH, Berlin, Germany; 2 InGef – Institute for Applied Health Research Berlin GmbH, Berlin, Germany; 3 Novartis Pharma GmbH, Nuremberg, Germany; 4 Frank Andersohn Consulting & Research Services, Berlin, Germany; 5 University Hospital Halle, Halle (Saale), Germany

**Keywords:** Polycythemia vera, Epidemiology, Claims data analysis, Cytoreductive therapy, Thromboembolic events, Germany

## Abstract

There is little evidence, particularly in Germany, on the epidemiology and the cytoreductive management of polycythemia vera (PV). We performed an observational study based on anonymized health claims data to provide estimates of the epidemiology of PV in Germany, to describe the use of cytoreductive drugs in patients with PV, and to assess the occurrence of thromboembolic events (TEs) in prevalent patients on continuous treatment with relevant cytoreductive drugs over time. For the year 2021, we estimated a PV prevalence of 28.6 per 100,000 and an incidence of 3.3 per 100,000 in the German adult population (≥ 18 years). We identified 83.2% of prevalent patients in 2021 as being at high risk for thromboembolic complications, based on age (≥ 60 years) and/or history of TEs. Contrary to treatment guidelines, 43.6% of these high-risk patients did not receive cytoreductive drug treatment in 2021. 63.5% of patients in 2021 who were treated with hydroxyurea (but not ruxolitinib) in that year, met our defined proxy criteria for intolerance/resistance to hydroxyurea. Over time, we observed a lower proportion of patients with TEs in patients continuously treated with ruxolitinib compared to patients treated with hydroxyurea who also met our defined proxy criteria for intolerance/resistance to hydroxyurea (35.8% vs. 56.3% after three years). Our findings suggest that currently available cytoreductive therapies are not being fully utilized according to treatment guidelines, which may lead to avoidable thromboembolic complications in this patient population.

**Supplementary Information:**

The online version contains supplementary material available at 10.1007/s00277-025-06192-6.

## Background

Polycythemia vera (PV) is a Philadelphia chromosome-negative myeloproliferative neoplasm characterized by an overproduction of red blood cells, along with an elevated white blood cell and platelet production and an increased risk of thromboembolic events (TEs) [1]. Comorbid cardiovascular risk factors may further increase the risk of TEs and have been associated with worse survival in patients with PV [2]. PV primarily affects older individuals, with a median age at diagnosis of 65 years [3]. Over time, PV may progress to myelofibrosis and in rare instances to acute myeloid leukemia (AML) [4]. Incidence estimates of PV in the US and Europe range from 0.4 to 2.8 per 100,000 with a slightly higher incidence reported in men than in women [3, 5–7]. To our knowledge, epidemiologic data specifically for Germany are currently not available.

There is currently no cure for PV, thus treatment is aimed at reducing the risk of TEs, managing clinical symptoms, and preventing complications. Initial treatment typically involves phlebotomy therapy in combination with low-dose acetylsalicylic acid to lower the hematocrit - the volume of red blood cells in the blood. Subsequent therapy decisions are mainly influenced by the patient’s individual risk for thrombosis, with older age and previous thrombosis being main risk factors. According to treatment guidelines, patients aged 60 years or older, or those with a history of arterial or venous thrombosis, are classified as high-risk patients [8, 9]. Cytoreductive therapy (hydroxyurea [HU] or interferon [IFN]-alfa) is recommended for high-risk patients and for low-risk patients in some circumstances (e.g., need of frequent phlebotomy therapy).

A change of therapy is indicated if patients develop intolerance or resistance to the primary therapy, or when severe clinical symptoms remain uncontrolled. Depending on the cytoreductive first-line therapy, possible second-line therapies include HU, IFN-alfa, ruxolitinib (RUX), or busulfan, an off-label option for patients of advanced age if no other treatment option is available [8]. HU is a common first-line treatment of high-risk PV [8, 10]. However, previous studies have shown that effectiveness can vary, and some patients develop intolerance or resistance [11, 12]. Resistance to HU has been linked to poorer outcomes including shorter survival and higher risk of disease transformation [11]. The European LeukaemiaNet (ELN) has proposed consensus criteria for intolerance/resistance, which include inadequate control of hematocrit, platelet count, and leukocyte count, persistent severe or distressing disease-related symptoms (e.g., pruritus), occurrence of bleeding, hematologic and non-hematologic toxicity, and development of non-melanotic skin cancer [9, 13].

In 2015, the European Medicines Agency (EMA) approved the JAK1/JAK2 inhibitor RUX for adult patients with PV who are HU-intolerant/resistant [14]. Clinical studies demonstrated RUX’s superiority over the best available therapy, offering better symptom control, reduced risk of TEs, and longer thromboembolic event-free survival [15–17].

Treatment of PV predominantly occurs in the outpatient setting. However, published data on the treatment of patients with PV with cytoreductive drugs often originate from clinical trials and university hospital settings, thus limiting insights into real-world treatment. Observational data detailing routine PV management is scarce.

We performed an analysis based on German health claims data with two aims. First, we wanted to provide estimates of the epidemiology of PV in Germany, characterize the prevalent patient population in terms of risk status and comorbidity burden, including comorbid cardiovascular risk factors (CVRFs), and provide a snapshot of the current treatment with cytoreductive drugs. Second, in a longitudinal analysis, we aimed to assess the occurrence of thromboembolic events (TEs) over time, focusing on patients on continuous treatment with cytoreductive medicines.

## Methods

### Study design, data source, and observation periods

We conducted this study in a retrospective, non-interventional cohort design. We used anonymized health claims data from the sample database of the Institute for Applied Health Research Berlin (InGef). This data set contains anonymized health claims data from a total of about four million persons insured by the German statutory health insurance (SHI) and is considered representative for the German population in terms of age and sex [18]. The data provides individual-level information across different healthcare sectors over time, covering sociodemographic information as well as information on hospitalizations (including dates of hospital admission and discharge, diagnostic and therapeutic procedures, main and secondary diagnoses), outpatient services (including diagnoses, diagnostic and therapeutic procedures), and outpatient drug prescriptions (including dates of prescription by a physician and dispensation by a pharmacy). All diagnoses in the database are coded according to the German modification of the 10th version of the International Classification of Diseases (ICD-10-GM). Outpatient diagnoses, which also include information on the diagnostic certainty (confirmed, suspected, diagnoses ruled out, and post-diagnosis status), are only recorded quarterly (i.e., four three-month periods per calendar year) in line with the quarterly reimbursement. Outpatient drug prescriptions are coded according to Anatomical Therapeutic Chemical (ATC) codes. Outpatient diagnostic and therapeutic procedures are coded according to doctor’s fee scale (“Einheitlicher Bewertungsmaßstab”, EBM) numbers while inpatient diagnostic and therapeutic procedures are coded according to the German procedure classification (“Operationen- und Prozedurenschlüssel”, OPS) codes.

We analyzed health claims data from January 1, 2014 to December 31, 2022, the most recent available data years available at the time of the study. To apply exclusion and validation criteria for prevalent and incident case identification, the data years 2014 and 2015 served as pre-observation period and 2022 as post-observation period. Results are reported for the period from January 1, 2016 to December 31, 2021.

We estimated the one-year prevalence and the one-year incidence of PV for the calendar years 2016 to 2021. For the year 2021, we additionally described prevalent patients in terms of cytoreductive treatment, specifically examining the proportion of patients with at least one dispensation of pre-defined cytoreductive drugs, and in terms of comorbidities including CVRFs, specifically examining the proportion of patients with at least one pre-defined diagnosis of the respective disease in comparison to a matched control population (selection described below).

In addition, we analyzed the occurrences of thromboembolic events over consecutive half-year periods for up to three years, focusing on patients who had been consistently receiving cytoreductive treatment with either HU or RUX. These patients were categorized into distinct cohorts based on their treatment, with the detailed criteria for cohort selection described below.

### Study population

The study population included all insured persons aged 18 years and older who were continuously insured in the two years before the respective reference year, and who were either continuously insured or continuously insured until death during the respective reference year. All populations considered were identified from this population.

### Identification of patients with prevalent PV

Individuals were classified as having prevalent PV if they met the following criteria in the respective reference year:

(i) at least one diagnosis of PV (ICD-10-GM code: D45.-) as a main or secondary hospital diagnosis or as a confirmed outpatient diagnosis;
(ii) no diagnosis of osteomyelofibrosis (D47.4), essential (hemorrhagic) thrombocythemia (D47.3), other myeloproliferative neoplasms (C92.1, D47.0, D47.1, D47.5), or other erythrocytosis (D75.0, D75.1) as a hospital main diagnosis or as a diagnosis by a hematology specialist in the outpatient sector.

- In addition, to validate prevalent case identification, at least one of the following criteria had to be met either in the respective reference year or the two preceding calendar years:

(i) dispensation of a cytoreductive drug (HU or IFN alfa);
(ii) main hospital diagnosis of PV;
(iii) confirmed outpatient diagnosis of PV by a hematology specialist;
(iv) main hospital diagnosis of a TE and a secondary hospital diagnosis of PV during the same hospitalization;
(v) bone marrow puncture performed in the outpatient sector;
(vi) phlebotomy performed in the outpatient sector.

Cytoreductive drugs dispensed by the pharmacy were identified by documented ATC codes, and inpatient administration of cytoreductive drugs was identified by OPS codes. Outpatient procedures (bone marrow puncture and phlebotomy) were identified by documented EBM numbers (for the operationalization see Table S1 in the supplement). TEs were identified by documented ICD-10-GM codes (see Table S2 in the supplement for operationalization).

### Identification of patients with incident PV

Individuals were classified as having incident PV if they met the following criteria:

(i) at least one diagnosis of PV as a main or secondary hospital diagnosis or as a confirmed outpatient diagnosis by a hematology specialist in the respective reference year;
(ii) no diagnosis of PV as a main or secondary hospital diagnosis or as a confirmed outpatient diagnosis in the two years prior to the first observable diagnosis of PV;
(iii) no diagnosis of osteomyelofibrosis, essential (hemorrhagic) thrombocythemia, other myeloproliferative neoplasms, or other erythrocytosis by a hematology specialist or as a main hospital diagnosis in the two years prior to the first observable diagnosis of PV.

In addition, at least one of the following criteria had to be met to validate incident case identification:

(i) dispensation of a cytoreductive drug (HU or IFN alfa) in the year after the first observable diagnosis of PV,
(ii) main hospital diagnosis of a TE and a secondary hospital diagnosis of PV during the same hospitalization in the year after the first observable diagnosis of PV;
(iii) bone marrow puncture performed in the outpatient sector within three months before or after the first observable diagnosis of PV;
(iv) phlebotomy therapy in the year after the first observable PV diagnosis.

### Estimation of prevalence and incidence

To estimate the one-year prevalence of PV, we divided the number of patients with prevalent PV in a given reference year by the study population in that year. Similarly, to determine the one-year incidence, we divided the number of patients with incident PV in a given reference year by the study population in that year. We then extrapolated the prevalence and incidence estimates to the entire German adult population (≥ 18 years) using direct standardization (by age and sex) based on population estimates for the respective reference year from the German Federal Statistical Office [19].

### Characteristics and treatment with cytoreductive drugs of prevalent patients in 2021

For 2021, we stratified prevalent patients according to risk status. Patients with a TE between 2019 and 2021, and those aged 60 years and older in 2021, were classified as high-risk PV patients. The remaining patients were classified as low-risk PV patients.

We further stratified the prevalent patients in 2021 based on the cytoreductive treatment they received in that year. This was done by assessing the proportion of patients who received specific cytoreductive drugs from pharmacies, using disjunctive strata. Among prevalent patients in 2021, we identified those who had at least one dispensation of HU but no dispensation of RUX. Within this group, we identified those patients with signs of HU intolerance/resistance (operationalization described below).

Next, we analyzed the prevalence of CVRFs and other comorbid conditions among the prevalent patients and their various strata. Results were further compared to a matched control population of insured persons without PV. The control population was selected from a pool of individuals within the study population in 2021 who did not meet the prevalent PV case definition and who did not have a diagnosis of osteomyelofibrosis, essential (hemorrhagic) thrombocythemia, other myeloproliferative neoplasms, or other erythrocytosis (as a hospital main diagnosis or as a diagnosis by a hematology specialist in the outpatient sector). The control population was matched to the prevalent patient population in 2021 at a ratio of 10:1 with respect to similar age (age categories: 18–24, 25–29, 30–34, …, 75–79, 80–84, ≥ 85), same sex, and presence of diagnoses of the same CVRF(s) (considering diabetes, obesity, hypercholesterolemia, smoking, and/or arterial hypertension) in 2021 (for operationalization see Table S3 in the supplement).

The prevalence of CVRFs, major bleeding events, and other comorbid conditions was assessed by determining the proportion of prevalent patients and controls with documented diagnoses for specific diseases according to pre-defined ICD-10-GM codes as a main or secondary hospital diagnosis or as a confirmed outpatient diagnosis in 2021 (for operationalization see Table S3 in the supplement). A single diagnosis code was sufficient to identify the respective disease.

### HU intolerance/resistance criteria

Most consensus criteria for HU intolerance/resistance rely on clinical parameters that are not available in German health claims data. Therefore, we identified affected patients using proxy measures. We used ICD-10-GM diagnosis codes (as main or secondary hospital diagnosis or as confirmed outpatient diagnosis), mostly including diagnoses that may indicate non-hematologic toxicity, and the EBM therapy number for phlebotomy, which may indicate inadequate hematocrit control (see Table 1 for operationalization). We classified patients as HU intolerant/resistant if they had any of the defined ICD-10-GM codes/EBM number in the same quarter of the year they received HU. This classification was only considered if the patient did not receive RUX during the same period.

**Table 1:** Guideline criteria for intolerance/resistance to HU and operationalization of these criteria in this study by proxy using health claims

	Operationalization in this study by proxy based on ICD-10-GM diagnosis codes and EBM numbers			Guideline definition of HU intolerance/resistance^1^
	Code type	Code(s)	Description	1. Need for phlebotomy to keep haematocrit < 45% after 3 months of at least 2 g/day of HU, OR 2. Uncontrolled myeloproliferation, i.e. platelet count > 400 × 10^9^ /l AND white blood cell count > 10 × 10^9^/l after 3 months of at least 2 g/day of HU, OR 3. Failure to reduce massive (organ extending by more than 10 cm from the costal margin) splenomegaly by more than 50% as measured by palpation, OR failure to completely relieve symptoms related to splenomegaly, after 3 months of at least 2 g/day of HU, OR 4. Absolute neutrophil count < 1.0 × 10^9^/l OR platelet count < 100 × 10^9^/l or haemoglobin < 100 g/l at the lowest dose of HU required to achieve a complete or partial clinicohaematological response, OR 5. Presence of leg ulcers or other unacceptable HU-related non-haematological toxicities, such as mucocutaneous manifestations, gastrointestinal symptoms, pneumonitis or fever at any dose of HU.
Intolerance	ICD-10-GM	J70.2	Acute drug-induced interstitial lung disorders	
	ICD-10-GM	J70.3	Chronic drug-induced interstitial lung disorders	
	ICD-10-GM	J70.4	Drug-induced interstitial lung disorders, unspecified	
	ICD-10-GM	L97	Ulcer of lower limb, not elsewhere classified	
	ICD-10-GM	L27.0	Generalized skin eruption due to drugs and medicaments	
	ICD-10-GM	L27.1	Localized skin eruption due to drugs and medicaments	
	ICD-10-GM	Y57.-!	Drugs and medicines causing adverse effects in therapeutic use	
	ICD-10-GM	L53.-	Other erythematous conditions	
	ICD-10-GM	L53.0	Toxic erythema	
	ICD-10-GM	M33.1	Other dermatomyositis	
	ICD-10-GM	M31.-	Other necrotizing vasculopathies	
	ICD-10-GM	L81.-	Other disorders of pigmentation	
	ICD-10-GM	L81.4	Other melanin hyperpigmentation	
	ICD-10-GM	K12.3	Oral mucositis (ulcerative)	
	ICD-10-GM	K29.-	Gastritis and duodenitis	
	ICD-10-GM	K52.-	Other noninfective gastroenteritis and colitis	
	ICD-10-GM	L57.0	Actinic keratosis	
	ICD-10-GM	C43.-	Malignant melanoma of skin	
	ICD-10-GM	C44.-	Other malignant neoplasms of skin	
	ICD-10-GM	L20-L30	Dermatitis and eczema	
	ICD-10-GM	L40-L45	Papulosquamous skin diseases	
	ICD-10-GM	L50-L54	Urticaria and erythema	
	ICD-10-GM	L81.-	Other disorders of pigmentation	
	ICD-10-GM	L95.-	Vasculitis limited to skin, not elsewhere classified	
	ICD-10-GM	L97.-	Ulcer of lower limb, not elsewhere classified	
	ICD-10-GM	L63.-	Alopecia areata	
	ICD-10-GM	L65.-	Other nonscarring hair loss	
Resistance	EBM	13,505	Phlebotomy	
	ICD-10-GM	L29.-	Pruritus	

^1^ [13]

HU – hydroxyurea, EBM - doctor’s fee scale, ICD-10-GM - German modification of the 10th version of the International Classification of Diseases

HU – hydroxyurea, PV - polycythemia vera

*thromboembolic event between 2019–2021 or aged ≥ 60 years in 2021

### Formation of treatment cohorts

To analyze the prevalence of comorbid CVRFs and the occurrence of thromboembolic events over time, we formed multiple treatment cohorts. Patients with prevalent PV who continuously received treatment with HU or RUX between 2016 and 2020 were included in the ‘HU cohorts’ or ‘RUX cohorts’, respectively. To be included in the HU cohorts, patients could not have been dispensed any RUX during the assessed period. Cohort inclusion was independent of previous treatment status, meaning both new and prevalent HU-treated patients were included. The index date was the first observable dispensation of HU or RUX.

A treatment episode was considered ‘continuous’ if HU or RUX was reimbursed subsequently at least twice in 182-day-periods. Patients were observed during the continuous treatment episode.

Within the HU cohorts, sub-cohorts of HU-treated patients for whom we could observe signs of HU intolerance/resistance (as previously defined) in the same quarter of an HU dispensation were selected (‘HU_IT/R_ cohorts’).

Cohorts of varying treatment durations were selected based on the number of consecutive 182-day-periods with documented continuous treatment (364 days [two 182-day-periods], 546 days [three 182-day-periods], … 1,092 days [six 182-day-periods]). In total, 15 treatment cohorts were formed: 5 RUX cohorts, 5 HU cohorts, and 5 HU_IT/R_ cohorts. The cohorts were observed for approximately 1 to 3 years.

Patients could be included in all cohorts for which they met the criteria and could potentially be included in multiple cohorts if they had multiple continuous treatment episodes between 2016 and 2020.

### Statistical analyses

In descriptive analyses, we summarized categorical variables by frequency and percentage, while continuous variables are presented as mean ± standard deviation (SD).

Furthermore, we compared results between groups (prevalent patients vs. matched control population; RUX cohorts vs. HU cohorts; RUX cohorts vs. HU_IT/R_ cohorts) using simple significance tests (depending on sample sizes, chi-square test or Fisher’s exact test for categorical variables and t-tests for continuous variables).

All analyses were performed using R Statistical Software. P-values < 0.05 were considered statistically significant and were not adjusted for multiplicity.

## Results

### Prevalence and incidence

**Fig. 1:**
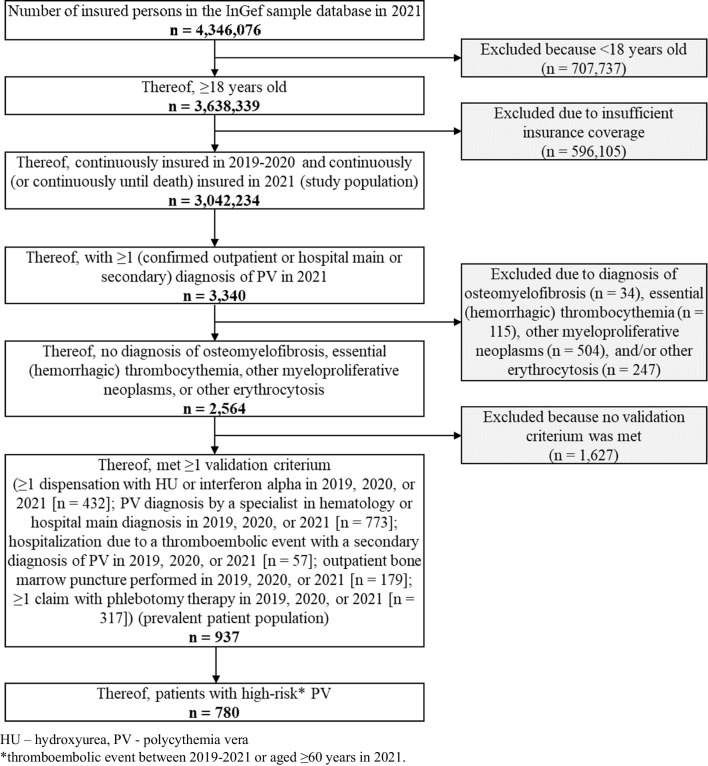
Selection steps for the prevalent patient population in 2021

We identified 937 patients with prevalent PV in the database in 2021 (Fig. 1), corresponding to a one-year prevalence of 30.8 per 100,000. When extrapolated to the German population, this equated to *n* = 19,805 and a prevalence of 28.6 per 100,000 (Table 2). The prevalence was higher in men than women (33.6 per 100,000 vs. 22.1 per 100,000) and increased with age, peaking in those 80 years and older (81.3 per 100,000). A total of 780 (83.2%) patients met our defined criteria for high-risk PV (i.e., TE between 2019 and 2021 or age 60 or older in 2021). In 2021, we identified 107 patients with incident PV in the database, corresponding to a one-year incidence of 3.5 per 100,000. When extrapolated to the German population, this equated to *n* = 2,286 and an incidence of 3.3 per 100,000 (Table 3). Figure S1 in the supplement shows the selection steps for incident patients in 2021.

**Table 2:** Age- and sex-stratified one-year prevalence of polycythemia vera (PV) in 2021

Age	Women		Men		All	
	*n* prevalent cases in the InGef sample database	Prevalence per 100,000	*n* prevalent cases in the InGef sample database	Prevalence per 100,000	*n* prevalent cases in the InGef sample database	Prevalence per 100,000
18–39 years	5	1.21	27	6.01	32	3.70
40–49 years	15	6.98	35	15.94	50	11.51
50–59 years	55	19.48	70	24.60	125	22.05
60–69 years	81	32.62	127	53.94	208	43.00
70–79 years	141	68.78	121	72.05	262	70.25
≥ 80 years	141	73.33	119	93.38	260	81.32
All (≥ 18 years)	438	28.12	499	33.61	937	30.80
Extrapolation to the German population	7,502	22.10	12,303	34.72	19,805	28.55

**Table 3:** Estimated prevalence and incidence of polycythemia vera (PV) in the German population (2016–2021)

Year	Prevalence		Incidence	
	*n* prevalent cases (extrapolated)	Prevalence per 100,000	*n* incident cases (extrapolated)	Incidence per 100,000
2016	17,610	25.50	931	1.35
2017	18,299	26.42	920	1.33
2018	17,766	25.59	633	0.96
2019	19,286	27.75	1,263	1.82
2020	20,534	29.58	2,495	3.59
2021	19,805	28.55	2,286	3.30

Extrapolated to the German population, we estimated that over 17,000 adult persons were affected by PV in Germany each year from 2016 to 2021. This resulted in a prevalence estimate ranging from 25.5 per 100,000 (in 2016) to 29.6 per 100,000 (in 2020) (Table 3). The estimated number of patients with incident PV in the adult German population and the corresponding incidence estimates were higher from 2019 to 2021 compared to our estimates from 2016 to 2018 (Table 3).

### Cytoreductive treatment among prevalent patients in 2021

**Fig. 2:**
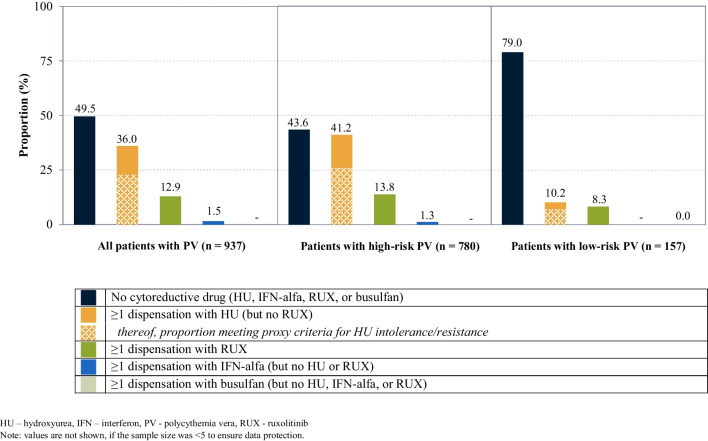
Cytoreductive treatment of prevalent patients in 2021, stratified by risk group

In 2021, nearly half of the prevalent patients (*n* = 464; 49.5%) did not receive a cytoreductive drug. 337 patients (36.0%) received HU at least once (but no RUX). Among these, 63.5% (*n* = 214) met our defined criteria for HU intolerance/resistance (Fig. 2). Fewer patients received RUX (*n* = 121; 12.9%). IFN-alfa (*n* = 14; 1.5%) and busulfan (*n* < 5) played minor roles.

Among patients with high-risk PV, 43.6% (340 out of 780 patients) received no cytoreductive drug. Another 321 (41.2%) received HU at least once (but no RUX); of these, 63.2% (*n* = 203) met our defined criteria for HU intolerance/resistance.

### Cardiovascular risk factors and comorbidity burden in prevalent patients in 2021

In 2021, the majority of prevalent patients (*n* = 802; 85.6%) had at least one comorbid CVRF (Table 4). The most common CVRFs were arterial hypertension (75.7%), followed by hypercholesterolemia/dyslipidemia (42.5%), and diabetes (25.4%).

**Table 4:** Cardiovascular risk factors, stratified by risk group and cytoreductive treatment, 2021

	Patients with prevalent PV (*n* = 937)		Subgroups											
			High-risk patients (*n* = 780)		Low-risk patients (*n* = 157)		Patients without cytoreductive drug dispensation (HU, IFN-alfa, RUX, or busulfan) (*n* = 464)		Patients with ≥ 1 HU dispensation, but no RUX dispensation				Patients with ≥ 1 RUX dispensation (*n* = 121)	
									Signs of HU resistance/intolerance (*n* = 214)		No signs of HU resistance/intolerance (*n* = 123)			
**Cardiovascular risk factors**	**n**	**%**	**n**	**%**	**n**	**%**	**n**	**%**	**n**	**%**	**n**	**%**	**n**	**%**
≥ 1 CVRF	802	85.6	699	89.6	103	65.6	400	86.2	183	85.5	104	84.6	104	86.0
Arterial hypertension	709	75.7	633	81.2	76	48.4	341	73.5	165	77.1	97	78.9	96	79.3
Hypercholesterolemia/dyslipidemia	398	42.5	362	46.4	36	22.9	195	42.0	104	48.6	50	40.7	45	37.2
Diabetes	238	25.4	216	27.7	22	14.0	136	29.3	55	25.7	26	21.1	19	15.7
Obesity	184	19.6	157	201	27	17.2	112	24.1	37	17.3	15	12.2	19	15.7
Smoking	128	13.7	92	11.8	36	22.9	94	20.3	21	9.8	9	7.3	< 5	-

CVRF - Cardiovascular Risk Factor, HU – hydroxyurea, IFN – interferon, PV - polycythemia vera, RUX - ruxolitinib

Comorbid CVRFs were more common among high-risk patients, with 89.6% having at least one CVRF, 81.2% with arterial hypertension, and 46.4% having diabetes.

Certain diseases were more common in patients with prevalent PV than the matched control population of insured persons without PV (Table 5; full results are presented in Table S4 in the supplement). The largest absolute differences were seen in hepatomegaly and splenomegaly (14.9% vs. 1.1%; *p* < 0.0001), kidney failure (22.8% vs. 15.2%; *p* < 0.0001), and heart failure (19.9% vs. 12.9%; *p* < 0.0001). Additionally, non-melanoma skin cancer was also more common in patients with PV than in controls (10.1% vs. 5.5%; *p* < 0.0001).

**Table 5:** Selected diagnoses in patients with prevalent polycythemia vera (PV) compared to the control population, 2021

Diagnosis	Patients with prevalent PV (*n* = 937)		Control population (*n* = 9,370)		*p*-value
	*n*	%	*n*	%	
Thromboembolic event	307	32.8	1,703	18.2	< 0.0001
Chronic ischemic heart disease	224	23.9	1,815	19.4	0.0009
Depression	218	23.3	1,886	20.1	0.0231
Kidney failure	214	22.8	1,425	15.2	< 0.0001
Heart failure	186	19.9	1,212	12.9	< 0.0001
Atrial fibrillation	177	18.9	1,260	13.4	< 0.0001
Chronic obstructive pulmonary disease	165	17.6	1,264	13.5	0.0005
Eczema	151	16.1	1,276	13.6	0.0348
Other diseases of the liver	149	15.9	1,184	12.6	< 0.0001
Hepatomegaly and splenomegaly, not elsewhere classified	140	14.9	102	1.1	< 0.0001
Hypertensive heart disease	137	14.6	937	10.0	< 0.0001
Urinary tract infection, localization unspecified	105	11.2	696	7.4	< 0.0001
Iron deficiency anemia, iron deficiency	103	11.0	444	4.7	< 0.0001
Sleep apnea	97	10.4	640	6.8	0.0001
Other malignant neoplasms of skin	95	10.1	520	5.5	< 0.0001
Edema	81	8.6	514	5.5	0.0001
Major bleeding event	54	5.8	336	3.6	0.0009
Cystitis	53	5.7	304	3.2	0.0001

PV - polycythemia vera

TEs (32.8% vs. 18.2%; *p* < 0.0001) were also more frequently observed in patients with PV than in matched controls (Table 5). Among high-risk patients, 39.4% had at least one TE in 2021 (results shown in Table S5 in the supplement). A higher proportion of high-risk patients who received a cytoreductive drug had at least one TE in 2021 compared to those who did not receive a cytoreductive drug (41.4% vs. 36.8%, respectively).

**Fig. 3:**
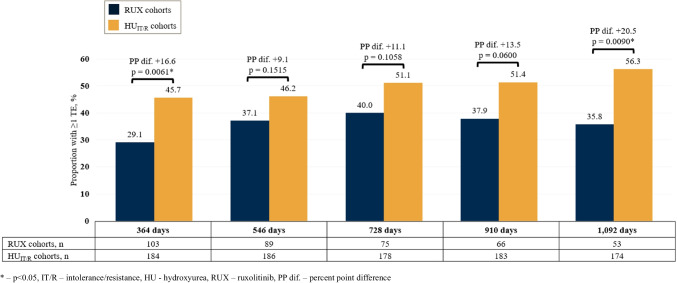
Patients with ≥1 thromboembolic event (TE) in the respective observation period

### Cardiovascular risk factors and thromboembolic events over time

Cohort sizes and characteristics for the HU_IT/R_ cohorts and RUX cohorts are shown in Table 6 (cohort sizes, characteristics, and results regarding the occurrence of TEs over time for the HU cohorts are shown in Table S6 in the supplement). Compared to patients in the HU_IT/R_ cohorts, patients in the RUX cohorts were on average between 4.9 (74.0 vs. 69.1 years; 546-day cohorts) to 6.6 (74.9 vs. 68.3 years; 910-day cohorts) years younger. For each observation period, the proportion of patients with 2 and ≥ 3 CVRFs was higher in the HU_IT/R_ cohorts than in the RUX cohorts. Cohorts with longer observation periods tended to have a higher proportion of patients with 2 and ≥ 3 CVRFs.

**Table 6:** Cardiovascular risk factors (CVRFs) in the treatment cohorts over time

		HU_IT/*R*_ cohorts (Patients on continuous Tx with HU who met proxy criteria for HU intolerance/resistance)					RUX cohorts (Patients on continuous Tx with RUX)				
Observation period, days		364	546	728	910	1,092	364	546	728	910	1,092
Cohort size, n		184	186	178	183	174	103	89	75	66	53
Female, %		53.3	54.3	53.9	56.3	56.9	45.6	46.1	48.0	45.5	50.9
Age, Mean ± SD		74.6 ± 9.5	74.0 ± 10.0	74.7 ± 9.7	74.9 ± 9.3	74.6 ± 9.5	68.9 ± 11.0	69.1 ± 11.3	68.4 ± 11.2	68.3 ± 10.8	68.1 ± 10.8
Number of CVRFs, %	0	13.0	14.0	10.7	9.3	9.8	19.4	14.6	14.7	13.6	11.3
	1	39.7	36.6	36.0	31.1	29.3	48.5	51.7	49.3	45.5	45.3
	2	25.5	26.9	28.7	33.9	33.3	18.4	22.5	25.3	28.8	26.4
	≥ 3	21.7	22.6	24.7	25.7	27.6	13.6	11.2	10.7	12.1	17.0
Diabetes, %		21.7	20.4	20.8	23.0	24.1	17.5	18.0	12.0	15.2	15.1
Obesity, %		15.8	17.7	19.7	21.9	21.8	8.7	9.0	12.0	10.6	15.1
Hypercholesterolaemia/ dyslipidaemia, %		27.7	31.7	35.4	37.7	41.4	22.3	23.6	25.3	30.3	24.5
Smoking, %		6.0	5.9	5.1	4.9	5.7	-	-	-	-	-
Arterial hypertension, %		80.4	80.6	84.3	86.3	85.6	71.8	76.4	78.7	78.8	86.8

CVRF - Cardiovascular Risk Factor, HU - hydroxyurea, RUX – ruxolitinib, Tx – treatment, SD – standard deviation

In the 364-day cohorts, the proportion of patients with obesity was 7.1%-points higher in the HU_IT/R_ cohort than in the RUX cohort (15.8% vs. 8.7%). Similarly, the proportion of patients with hypertension was 8.6%-points higher in the HU_IT/R_ cohort compared to the RUX cohort (80.4% vs. 71.8%). However, in the 1,092-day cohorts, the proportion of patients with hypertension was slightly higher in the RUX cohort (86.8% vs. 85.6%). In contrast, the 1,092-day HU_IT/R_ cohort included a higher proportion of patients with diabetes (24.1% vs. 15.1%) and hypercholesterolemia/dyslipidaemia (41.4% vs. 24.5%) compared to the 1,092-day RUX-cohort.

For each observation period, TEs were more common in the HU_IT/R_ cohorts compared to the RUX cohorts (Fig. 3). In the 364-day RUX cohort, 29.1% of patients had at least one TE compared to 45.7% of patients in the 354-day HU_IT/R_ cohort (dif. 16.6%-points, *p* < 0.05). The absolute difference between the cohorts increased with the length of the observation periods and was highest for the 1,092-day observation period (dif. 20.5%-points, *p* < 0.05) (Fig. 3).

## Discussion

Based on a large representative German health claims database, we provide recent estimates of the prevalence and incidence of PV in the German adult population, and we characterize the prevalent patient population in 2021 in terms of risk status, comorbidity burden, and cytoreductive drug treatment. We further observed patients with PV on continuous cytoreductive treatment with HU or RUX for TEs over time.

Overall, there is little information on the epidemiology of PV available in the literature. In particular, data on the prevalence and incidence in Germany are lacking. Additionally, published studies on the prevalence of PV have yielded heterogeneous results. Based on peer-reviewed literature and online documentation from disease and health registries between 2000 and 2012, Moulard et al. estimated the prevalence of PV in the European Union at 4.96 to 30 per 100,000 [7]. Roaldsnes et al.‘s estimate of 9.2 per 100,000 based on Norwegian cancer registry data is at the lower end of this range [6], while the most recent Orphanet report appears to confirm the upper end of the range at 30.0 per 100,000 [20]. Our estimate for 2021 of 28.6 per 100,000 is also at the upper end of this range.

Published estimates on the incidence of PV in the US and Europe also vary, ranging from 0.4 to 2.8 per 100,000 [3, 5–7]. While our incidence estimates for the years 2016 to 2019 are within this range, our estimates for 2020 (3.59 per 100,000) and 2021 (3.30 per 100,000) are higher than those reported. The substantial increase in our estimate for 2020 is mainly due to methodological reasons, as we were only able to include phlebotomy therapy as a criterion to validate incident case identification from 2020 onwards. Phlebotomy (EBM number 13505) has only been billable since April 2020 – in addition to the basic fee – by specialists in internal medicine with a focus on hematology and internal oncology [21]. Thus, we suspect our incidence estimates for 2016 to 2019 and those reported in the literature to be underestimates, rather than our 2020 and 2021 estimates to be overestimates. Our prevalence estimates were also slightly higher in 2020 and 2021, when phlebotomy therapy could be included as a criterion to validate prevalent case identification. However, the difference was less pronounced, which indicates that our algorithm to identify prevalent patients worked well even without considering phlebotomy therapy. It should also be noted that phlebotomy therapy is a first-line treatment irrespective of risk status, thus a more pronounced effect was to be expected when including phlebotomy therapy as a validation criterion for newly diagnosed patients. As PV mainly affects individuals aged 60 and over [22, 23], an increasing trend in prevalence can be expected over the past decade and in the coming years due to changes in the age structure of the German population towards a higher proportion of persons of older age [24]. Increased awareness of PV among physicians may further exacerbate the increasing trend in prevalence in the future. Consistent with several studies [3, 5], we further found PV to be slightly more common in men than in women.

We considered most prevalent patients in 2021 (83.2%) to have high-risk PV, either due to their age (≥ 60 years in 2021) or their recorded history of TEs (criterion checked between 2019 and 2021), which is in line with previous observational studies in which the majority of patients were categorized as high-risk, mainly due to their age [22, 23]. Contrary to current treatment guideline recommendations [8, 9], 43.6% of the patints we categorized as high-risk patients in 2021 did not receive treatment with cytoreductive drugs in that year. This result represents a snapshot of the calendar year 2021, and we cannot rule out the possibility that patients started cytoreductive drug treatment in the following year. However, this finding is consistent with the results of a recent German chart review, which reported that on average only 60.7% of patients with high-risk PV received cytoreductive drug treatment. The authors further noted that the proportion of patients receiving cytoreductive drug treatment varied highly between treatment sites [22]. Next to the control of symptoms, prevention of TEs is the main aim of treatment in PV, as complications from TEs are the most frequent clinical challenge in patients with PV [25, 26]. In this context, much emphasis has been placed on control of the hematocrit below 45% as this target level has been associated with significantly lower rates of major TEs and death [27, 28]. Currently, there is strong consensus that patients with high-risk PV should be treated with cytoreductive drugs to reduce TE risk by controlling blood cell count [9, 27]. While we did observe a lower relative frequency of patients with TE in 2021 in high-risk patients who did not receive cytoreductive treatment compared to high-risk patients who received cytoreductive treatment in that year, it should be noted that due to the cross-sectional design it is not possible to determine the temporality of this finding; for example, patients may have been started on cytoreductive drug treatment only after the respective TE.

In line with previous observational studies [22, 29], the majority of patients with prevalent PV in 2021 (85.6%) had one or more comorbid CVRF(s), with arterial hypertension (75.7%) bein the most prevalent. Thus, it should be noted that most patients can be considered at elevated risk for cardiovascular disease by conventional risk categorization. Several recent studies have explored the association of CVRFs with different outcomes, including the occurrence of TEs, in patients with PV [2, 30, 31]. While CVRFs are currently not being considered for risk classification to inform the therapeutic decisions, the aggressive management of CVRFs is recommended for all patients [27].

Among others, we observed a higher occurrence of non-melanoma skin cancer in patients with PV than matched controls. Increased risk of non-melanoma skin cancer has previously been reported in patients with Philadelphia-negative myeloproliferative neoplasms, including PV, who are treated with HU and RUX [32]. While the underlying mechanisms and the extent to which cytoreductive drugs contribute to the increased risk are not yet fully understood, it has been suggested that prolonged treatment with HU may be a contributing factor [33]. Consequently, guidelines currently recommend regular dermatologic screenings both prior to and throughout the duration of these therapies [8].

In 2021, most prevalent patients on cytoreductive drug treatment received HU, but no RUX. Among these patients, we identified those with signs of HU intolerance/resistance. Since most consensus criteria for HU intolerance/resistance are based on clinical parameters that are not documented in German claims data, in particular constitutional symptoms, we identified affected patients by proxy using ICD-10-GM diagnosis codes, mostly for diagnoses that may indicate non-hematologic toxicity, and claims for phlebotomy, which may indicate inadequate hematocrit control. The operationalization based on documented diagnosis codes and claims for phlebotomy in our study was defined based on the originally proposed consensus criteria [13] in consultation with an expert. Nearly two-thirds (63.5%) of prevalent patients in 2021 who were treated with HU (but not RUX) met our defined proxy criteria for HU intolerance/resistance in that year. This finding is generally consistent with data from other observational studies showing that a considerable proportion of patients may not be adequately controlled with HU. In a prospective observational study of PV patients in the United States, 57% of patients treated with HU for more than three months continued to have an elevated hematocrit level above 45%, 33% continued to receive phlebotomies, and 27% experienced uncontrolled myeloproliferation [34]. In a cross-sectional study from Belgium, 60% of HU-treated patients had elevated values for hematocrit, platelet count, and/or white blood cell count [35]. Similarly, in a multicenter retrospective cohort study, 70% of patiens did not achieve complete response after 12 or more months of therapy with HU, of which 71.3% of patiens nevertheless continued treatment with HU [36].

Our results indicate that, contrary to guideline recommendations, there might be a number of patients whose treatment is not adjusted despite showing signs of potential HU resistance/intolerance. This raises concerns because intolerance and resistance to HU have been linked to adverse outcomes, including an increased risk of thrombosis, lower survival rates, and higher chances of disease progression [11, 37].

RUX has been approved by the European Medicines Agency in 2015 as a second-line therapy for the treatment of adults with PV who are resistant or intolerant to HU, and has shown favorable results in terms of disease control and thromboembolic event-free survival compared to best available therapy [15, 38, 39]. In our analysis, we observed a lower proportion of patients with TEs in patients continuously treated with RUX (RUX cohorts) compared to patients continuously treated with HU who also met our defined proxy criteria for HU intolerance/resistance (HU_IT/R_ cohorts). Some of these differences were statistically significant (1- and 3-year observation periods). However, these results should be interpreted as descriptive only. The analysis was not adjusted for differences in the composition of the cohorts (e.g., in terms age, CVRFs, treatment status [prevalent vs. new-user]). For example, due to small sample sizes, the treatment cohorts were not matched based on criteria known to increase the risk of TEs. Patients in the HU_IT/R_ cohorts were on average older and had more CVRFs than patients in the RUX cohorts, suggesting that younger and healthier patients may be more likely to receive RUX when treatment with HU is unsatisfactory.

### Limitations

We want to recognize the following limitations to our study. We considered patients 60 years and over and those with a history of TEs as patients with high-risk PV. However, the pre-observation period for identifying documented diagnoses for TEs was limited to two years. We may have misclassified patients because previous TEs may have been missed due to the short pre-observation period. Criteria indicating HU intolerance/resistance could only be indirectly operationalized by proxy because health claims data do not contain clinical parameters (e.g. haematocrit and symptoms) used in clinical practice to identify HU intolerance/resistance to HU. Comparisons between treatment cohorts were not adjusted for possible confounders, thus, results may be biased and should be interpreted in terms of real-world drug effectiveness. Furthermore, the treatment cohorts were observed for a maximum of three years, which is a relatively short period for patients with PV, for whom a median survival of 14 years after diagnosis has been reported [40].

## Conclusions

To our knowledge, this study is the first comprehensive analysis of the epidemiology of PV in Germany. Based on a large representative database we provide new insights into the characteristics of prevalent patients and their current real-life treatment situation. Our findings suggest that currently available cytoreductive therapies are not being fully utilized according to treatment guidelines. Because suboptimal treatment practices may increase the risk of thromboembolic complications in this patient population, further research, particularly studies including clinical data, are warranted to support the findings of this study that cytoreductive therapy in PV patients may not be fully in compliance with guidelines.

## Supplementary Information

Below is the link to the electronic supplementary material.

ESM 1 (DOCX 116 KB)

## Data Availability

The data analyzed in this study was retrieved from the Institute for Applied Health Research Berlin (InGef) Research Database and cannot be made available in the manuscript, the supplementary files, or in a public repository due to German data protection laws (Bundesdatenschutzgesetz). To facilitate the replication of results, anonymized data used for this study are stored on a secure drive at the InGef GmbH. Access to the data used in this study can only be provided to external parties under the conditions of the cooperation contract of this research project and can be assessed upon request, after written approval (contact: info@ingef.de), if required.
