# Supplementary material for: Prevalence, incidence, and thromboembolic events in polycythemia vera: a study based on longitudinal German health claims data
Source: Ann Hematol. 2025 Feb 10;104(1):347–60. doi: 10.1007/s00277-025-06192-6 (PMC11868326; doi:10.1007/s00277-025-06192-6)
Supplement: Supplementary file 1 — (DOCX 116 KB) [file 277_2025_6192_MOESM1_ESM.docx]

Table S1 Operationalization of relevant treatments

| **Treatment/**  **Procedure** | **Code type** | **Code** | **Description** |
| --- | --- | --- | --- |
| Bone marrow puncture | EBM | 02341 | Bone marrow puncture |
|  | EBM | 32163 | Bone marrow puncture |
|  | EBM | 32168 | Bone marrow puncture |
|  | EBM | 32169 | Bone marrow puncture |
| Phlebotomy | EBM | 13505 | Bloodletting with collection of at least 200 ml of blood |
| Interferon alfa | ATC-Code | L03AB04 | Interferon alfa-2a |
|  | ATC-Code | L03AB05 | Interferon alfa-2b |
|  | ATC-Code | L03AB10 | Peginterferon alfa-2b |
|  | ATC-Code | L03AB11 | Peginterferon alfa-2a |
|  | ATC-Code | L03AB15 | Ropeginterferon alfa-2b |
|  | OPS-Code | 8-812.1 | Interferon alfa-2a, parenteral |
|  | OPS-Code | 8-812.2 | Interferon alfa-2b, parenteral |
| Ruxolitinib | ATC-Code | L01EJ01 | Ruxolitinib |
|  | OPS-Code | 6-009.4 | Ruxolitinib |
| Hydroxyurea | ATC-Code | L01XX05 | Hydroxyurea |
| Busulfan | ATC-Code | L01AB01 | Busulfan |
|  | OPS-Code | 6-002.d | Busulfan |

ATC - Anatomical Therapeutic Chemical, EBM - doctor’s fee scale, OPS - German procedure classification (“Operationen- und Prozedurenschlüssel”)

Table S2 Operationalization of thromboembolic events

| **Thromboembolic event** | **ICD-10-GM code** |
| --- | --- |
| Acute myocardial infarction | I21.- |
| Pulmonary embolism | I26.- |
| Subarachnoid hemorrhage | I60.- |
| Intracerebral hemorrhage | I61.- |
| Other nontraumatic intracranial hemorrhage | I62.- |
| Cerebral infarction | I63.- |
| Stroke, not specified as hemorrhage or infarction | I64.- |
| Occlusion and stenosis of cerebral arteries, not resulting in cerebral infarction | I66.- |
| Other cerebrovascular diseases | I67.- |
| Sequelae of cerebrovascular disease | I69.- |
| Arterial embolism and thrombosis | I74.- |
| Phlebitis and thrombophlebitis | I80.- |
| Portal vein thrombosis | I81.- |
| Other venous embolism and thrombosis | I82.- |
| Transient cerebral ischemic attacks and related syndromes | G45.- |

Table S3 Operationalization of comorbidities, cardiovascular risk factors, and major bleeding events

| **Diagnosis** | **ICD-10-GM code** |
| --- | --- |
| Tuberculosis | A15-A19 |
| Zoster [herpes zoster] | B02.- |
| Hepatitis B (acute hepatitis B, chronic viral hepatitis B without delta-agent) | B16.-, B18.1- |
| Cryptococcosis | B45.- |
| Other malignant neoplasms of skin | C44.- |
| Iron deficiency anemia, iron deficiency | D50.0, D50.8, D50.9, E61.1 |
| Disorders of lipoprotein metabolism and other lipidemias | E78.- |
| Depression | F32-F33 |
| Encephalitis, myelitis and encephalomyelitis in viral diseases classified elsewhere | G05.1 |
| Multiple sclerosis | G35.- |
| Sleep apnea | G47.3 |
| Iridocyclitis, iridocyclitis in other diseases classified elsewhere | H20.-, H22.1 |
| Hypertensive heart disease | I11.- |
| Hypertensive renal disease | I12.- |
| Hypertensive heart and kidney disease | I13.- |
| Secondary hypertension | I15.- |
| Angina pectoris | I20.- |
| Recurrent myocardial infarction | I22.- |
| Certain current complications following acute myocardial infarction | I23.- |
| Other acute ischemic heart diseases | I24.- |
| Chronic ischemic heart disease | I25.- |
| Atrial fibrillation | I48.- |
| Heart failure | I50.- |
| Infections of the upper respiratory tract | J00-J06 |
| Flu and pneumonia | J09-J18 |
| Bronchitis, not specified as acute or chronic | J40.- |
| Chronic obstructive pulmonary disease | J42.-, J43.-, J44.- |
| Bronchial asthma | J45.- |
| Respiratory infections, not elsewhere classified | J98.7 |
| Peptic ulcer | K25-K28 |
| Inflammatory bowel disease (Crohn disease, ulcerative colitis) | K50.-, K51.- |
| Polyp of the colon | K63.5 |
| Chronic hepatitis, not elsewhere classified | K73.- |
| Fibrosis and cirrhosis of liver | K74.- |
| Other diseases of the liver | K76.- |
| Portal hypertension | K76.6 |
| Eczema | L20.-, L21.-, L23, L24, L25, L28, L30.- |
| Pruritus | L29.- |
| Psoriasis | L40.- |
| Rheumatoid arthritis | M05.-, M06.-, M08.-, M09.- |
| Psoriatic and enteropathic arthropathies | M07.- |
| Systemic lupus erythematosus | M32.- |
| Dermatopolymyositis | M33.- |
| Systemic sclerosis | M34.- |
| Other systemic involvement of connective tissue | M35.- |
| Ankylosing spondylitis | M45.- |
| Acute tubulo-interstitial nephritis | N10.- |
| Renal failure | N17-N19 |
| Cystitis | N30.- |
| Nonspecific urethritis | N34.1 |
| Urinary tract infection, localization unspecified | N39.0 |
| Hepatomegaly and splenomegaly, not elsewhere classified | R16.- |
| Edema | R60.- |
| **Cardiovascular risk factor** | **ICD-10-GM code** |
| Diabetes | E10-E14; G63.2 |
| Obesity | E66.- |
| Arterial hypertension | I10.- |
| Hypercholesterolemia/dyslipidemia | E78.0; E78.1; E78.2; E78.5 |
| Tobacco dependence | F17.-; T65.2 |
| **Major bleeding events** | **ICD-10-GM code/OPS-code** |
| Subarachnoid haemorrhage | I60.- |
| Intracerebral haemorrhage | I61.- |
| Other nontraumatic intracranial haemorrhage | I62.- |
| Multiple intracerebral and cerebellar haematomas | S06.23 |
| Focal cerebral haematoma | S06.33 |
| Focal cerebellar haematoma | S06.34 |
| Epidural haemorrhage | S06.4 |
| Nontraumatic spinal haemorrhage | G95.10 |
| Hyphaema | H21.0 |
| Choroidal haemorrhage and rupture | H31.3 |
| Retinal haemorrhage | H35.6 |
| Vitreous haemorrhage | H43.1 |
| Haemoperitoneum | K66.1 |
| Haemarthrosis | M25.0 |
| Haemopericardium, not elsewhere classified | I31.2 |
| Intramuscular bleeding with compartment syndrome | M62.2 + M62.8 |
| Whole blood transfusion, 1-5 units | 8-800.0 |
| Whole blood transfusion, more than 5 units | 8-800.1 |
| Transfusion of red cell concentrate | 8-800.c |
| Acute posthaemorrhagic anaemia | D62 |

ICD-10-GM - German modification of the 10th version of the International Classification of Diseases

Figure S1 Selection steps for the incident patient population in 2021


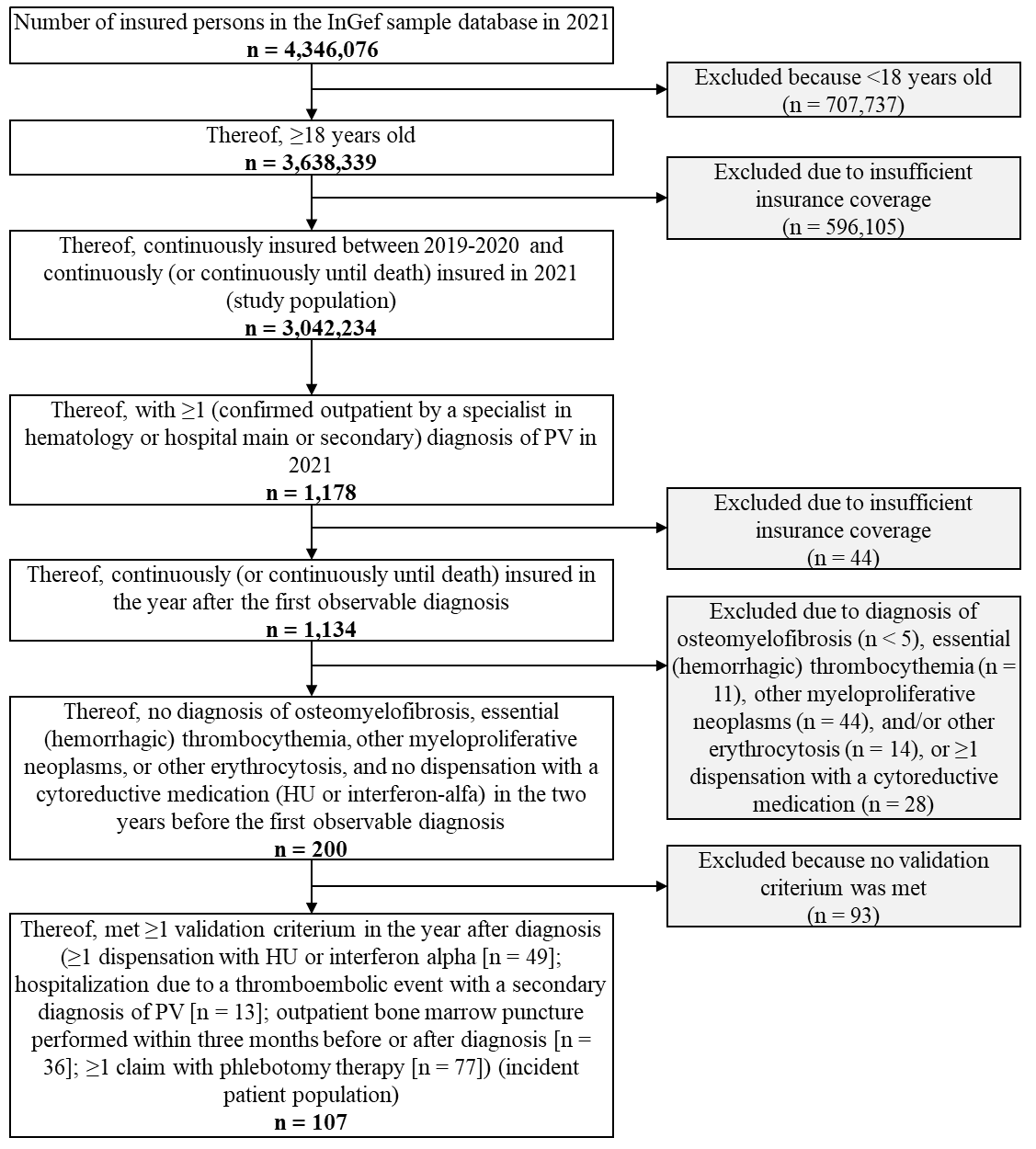


PV - polycythemia vera, HU – hydroxyurea

Table S4 Diagnoses in patients with prevalent polycythemia vera (PV) compared to the control population, 2021.

| **Diagnosis** | **Patients with prevalent PV**  **(n = 937)** | | **Control population**  **(n = 9,370)** | | **p-value** |
| --- | --- | --- | --- | --- | --- |
|  | **n** | **%** | **n** | **%** |  |
| Disorders of lipoprotein metabolism and other lipidemias | 425 | 45.4 | 4296 | 45.8 | 0.7737 |
| Thromboembolic event | 307 | 32.8 | 1,703 | 18.2 | <0.0001 |
| Chronic ischemic heart disease | 224 | 23.9 | 1815 | 19.4 | 0.0009 |
| Depression | 218 | 23.3 | 1886 | 20.1 | 0.0231 |
| Renal failure | 214 | 22.8 | 1425 | 15.2 | <0.0001 |
| Heart failure | 186 | 19.9 | 1212 | 12.9 | 0.0000 |
| Atrial fibrillation | 177 | 18.9 | 1260 | 13.4 | 0.0000 |
| Chronic obstructive pulmonary disease | 165 | 17.6 | 1264 | 13.5 | 0.0005 |
| Eczema | 151 | 16.1 | 1276 | 13.6 | 0.0348 |
| Other diseases of the liver | 149 | 15.9 | 1184 | 12.6 | 0.0045 |
| Hepatomegaly and splenomegaly, not elsewhere classified | 140 | 14.9 | 102 | 1.1 | <0.0001 |
| Hypertensive heart disease | 137 | 14.6 | 937 | 10.0 | 0.0000 |
| Infections of the upper respiratory tract | 108 | 11.5 | 1116 | 11.9 | 0.7289 |
| Urinary tract infection, localization unspecified | 105 | 11.2 | 696 | 7.4 | <0.0001 |
| Iron deficiency anemia, iron deficiency | 103 | 11.0 | 444 | 4.7 | <0.0001 |
| Sleep apnea | 97 | 10.4 | 640 | 6.8 | 0.0001 |
| Other malignant neoplasms of skin | 95 | 10.1 | 520 | 5.5 | <0.0001 |
| Bronchial asthma | 90 | 9.6 | 901 | 9.6 | 0.9916 |
| Edema | 81 | 8.6 | 514 | 5.5 | 0.0001 |
| Major bleeding event | 54 | 5.8 | 336 | 3.6 | 0.0009 |
| Cystitis | 53 | 5.7 | 304 | 3.2 | 0.0001 |
| Flu and pneumonia | 48 | 5.1 | 272 | 2.9 | 0.0002 |
| Psoriasis | 41 | 4.4 | 387 | 4.1 | 0.7195 |
| Pruritus | 36 | 3.8 | 202 | 2.2 | 0.0011 |
| Angina pectoris | 35 | 3.7 | 256 | 2.7 | 0.0771 |
| Other systemic involvement of connective tissue | 35 | 3.7 | 333 | 3.6 | 0.7754 |
| Peptic ulcer | 33 | 3.5 | 170 | 1.8 | 0.0003 |
| Rheumatoid arthritis | 32 | 3.4 | 361 | 3.9 | 0.5049 |
| Polyp of the colon | 31 | 3.3 | 340 | 3.6 | 0.6159 |
| Hypertensive heart and kidney disease | 20 | 2.1 | 88 | 0.9 | 0.0006 |
| Bronchitis, not specified as acute or chronic | 20 | 2.1 | 187 | 2.0 | 0.7729 |
| Hypertensive renal disease | 17 | 1.8 | 113 | 1.2 | 0.1116 |
| Secondary hypertension | 15 | 1.6 | 104 | 1.1 | 0.1798 |
| Zoster [herpes zoster] | 15 | 1.6 | 161 | 1.7 | 0.7914 |
| Inflammatory bowel disease (Crohn disease, ulcerative colitis) | 12 | 1.3 | 107 | 1.1 | 0.7047 |
| Fibrosis and cirrhosis of liver | 11 | 1.2 | 77 | 0.8 | 0.2639 |
| Iridocyclitis, iridocyclitis in other diseases classified elsewhere | 6 | 0.6 | 28 | 0.3 | 0.1221 |
| Respiratory infections, not elsewhere classified | 5 | 0.5 | 34 | 0.4 | n/a |
| Other acute ischemic heart diseases | 5 | 0.5 | 44 | 0.5 | n/a |
| Ankylosing spondylitis | 5 | 0.5 | 64 | 0.7 | n/a |
| Tuberculosis | <5 | - | 13 | 0.1 | n/a |
| Hepatitis B (acute hepatitis B, chronic viral hepatitis B without delta-agent) | <5 | - | 18 | 0.2 | n/a |
| Multiple sclerosis | <5 | - | 28 | 0.3 | n/a |
| Chronic hepatitis, not elsewhere classified | <5 | - | 18 | 0.2 | n/a |
| Portal hypertension | <5 | - | 11 | 0.1 | n/a |
| Psoriatic and enteropathic arthropathies | <5 | - | 36 | 0.4 | n/a |
| Systemic lupus erythematosus | <5 | - | 6 | 0.1 | n/a |
| Nonspecific urethritis | <5 | - | <5 | - | n/a |
| Encephalitis, myelitis and encephalomyelitis in viral diseases classified elsewhere | 0 | 0 | <5 | - | n/a |
| Recurrent myocardial infarction | 0 | 0 | <5 | - | n/a |
| Certain current complications following acute myocardial infarction | 0 | 0 | <5 | - | n/a |
| Cryptococcosis | 0 | 0.0 | 0 | 0.0 | n/a |
| Dermatopolymyositis | 0 | 0.0 | 8 | 0.1 | n/a |
| Acute tubulo-interstitial nephritis | 0 | 0.0 | 12 | 0.1 | n/a |
| Systemic sclerosis | 0 | 0.0 | 14 | 0.1 | n/a |

ICD-10-GM - German modification of the 10th version of the International Classification of Diseases

Note: values are not shown, if the sample size was <5 to ensure data protection

Table S5 Thromboembolic events stratified by risk group, 2021.

|  |  | | **Stratified according to risk group** | | | | | | | |
| --- | --- | --- | --- | --- | --- | --- | --- | --- | --- | --- |
| **Diagnosis** | **Patients with prevalent PV**  **(n = 937)** | | **High-risk patients**  **(n = 780)** | | **High-risk patients**  **(n = 780)** | | | | **Low-risk patients**  **(n = 157)** | |
|  |  |  |  |  | **With cytoreductive drug dispensation (n=440)** | | **No cytoreductive drug dispensation (n=340)** | |  |  |
|  | **n** | **%** | **n** | **%** | **n** | **%** | **n** | **%** | **n** | **%** |
| Any thromboembolic event | 307 | 32.8 | 307 | 39.4 | 182 | 41.4 | 125 | 36.8 | 0 | 0.0 |
| Acute myocardial infarction | 41 | 4.4 | 41 | 5.3 | 27 | 6.1 | 14 | 4.1 | 0 | 0.0 |
| Pulmonary embolism | 28 | 3.0 | 28 | 3.6 | 17 | 3.9 | 11 | 3.2 | 0 | 0.0 |
| Subarachnoid hemorrhage | <5 | - | <5 | - | <5 | - | <5 | - | 0 | 0.0 |
| Intracerebral hemorrhage | 6 | 0.6 | 6 | 0.8 | 5 | 1.1 | <5 | - | 0 | 0.0 |
| Other nontraumatic intracranial hemorrhage | <5 | - | <5 | - | <5 | - | 0 | 0.0 | 0 | 0.0 |
| Cerebral infarction | 62 | 6.6 | 62 | 8.0 | 43 | 9.8 | 19 | 5.6 | 0 | 0.0 |
| Stroke, not specified as hemorrhage or infarction | 37 | 4.0 | 37 | 4.7 | 25 | 5.7 | 12 | 3.5 | 0 | 0.0 |
| Occlusion and stenosis of cerebral arteries, not resulting in cerebral infarction | <5 | - | <5 | - | <5 | - | <5 | - | 0 | 0.0 |
| Other cerebrovascular diseases | 86 | 9.2 | 86 | 11.0 | 45 | 10.2 | 41 | 12.1 | 0 | 0.0 |
| Sequelae of cerebrovascular disease | 96 | 10.3 | 96 | 12.3 | 64 | 14.6 | 32 | 9.4 | 0 | 0.0 |
| Arterial embolism and thrombosis | 18 | 1.9 | 18 | 2.3 | 12 | 2.7 | 6 | 1.8 | 0 | 0.0 |
| Phlebitis and thrombophlebitis | 52 | 5.6 | 52 | 6.7 | 35 | 8.0 | 17 | 5.0 | 0 | 0.0 |
| Portal vein thrombosis | 9 | 1.0 | 9 | 1.2 | 8 | 1.8 | <5 | - | 0 | 0.0 |
| Other venous embolism and thrombosis | 32 | 3.4 | 32 | 4.1 | 16 | 3.6 | 16 | 4.7 | 0 | 0.0 |
| Transient cerebral ischemic attacks and related syndromes | 37 | 4.0 | 37 | 4.7 | 22 | 5.0 | 15 | 4.4 | 0 | 0.0 |

Notes: Patients with a TE between 2019 and 2021, and those aged 60 and older in 2021, were classified as high-risk PV. The remaining patients were classified as low-risk PV patients. Therefore, by definition, low-risk patients could not have experienced a TE in 2021. Values are not shown, if the sample size was <5 to ensure data protection

Table S6 Characteristics incl. cardiovascular risk factors (CVRFs) and thromboembolic events (TEs) in the HU cohorts over time.

|  | | **HU cohorts** | | | | |
| --- | --- | --- | --- | --- | --- | --- |
| **Observation period, days** | | **364** | **546** | **728** | **910** | **1,092** |
| Cohort size, n | | 359 | 325 | 292 | 276 | 251 |
| Female, % | | 55.2 | 54.8 | 54.1 | 54.3 | 55.0 |
| Age, Mean ±SD | | 73.4 ±10.4 | 73.2 ±10.3 | 73.2 ±10.2 | 73.2 ±10.2 | 72.8 ±10.2 |
| Number of CVRFs, % | 0 | 14.2 | 14.5 | 12.3 | 11.2 | 11.6 |
|  | 1 | 43.2 | 40.9 | 39.7 | 35.5 | 31.9 |
|  | 2 | 24.2 | 24.3 | 25.7 | 29.7 | 31.9 |
|  | ≥ 3 | 18.4 | 20.3 | 22.3 | 23.6 | 24.7 |
| Diabetes, % | | 20.1 | 19.7 | 19.9 | 20.7 | 22.3 |
| Obesity, % | | 13.9 | 15.4 | 17.4 | 20.3 | 19.9 |
| Hypercholesterolaemia/ dyslipidaemia, % | | 23.7 | 26.5 | 30.5 | 33.0 | 36.3 |
| Tobacco dependence, % | | 6.7 | 6.5 | 6.2 | 6.2 | 6.8 |
| Arterial hypertension, % | | 80.2 | 80.9 | 82.9 | 84.4 | 84.1 |
| Any TE, % | | 39.0 | 39.7 | 44.9 | 46.7 | 49.4 |
| Number of TEs, Mean ±SD | | 1.7 ±0.9 | 1.7 ±1.0 | 1.7 ±1.0 | 1.8 ±1.1 | 1.8 ±1.0 |

CVRF - Cardiovascular Risk Factor, HU – hydroxyurea , TE – thromboembolic event, SD – standard deviation

*p-value <0.05 compared to ruxolitinib-cohort

Note: values are not shown, if the sample size was <5 to ensure data protection
